# Comparative developmental competence of *in vitro* embryos recovered from Bali cattle with normal and poor sperm motility

**DOI:** 10.14202/vetworld.2024.593-601

**Published:** 2024-03-17

**Authors:** Hasbi Hasbi, Hikmayani Iskandar, Herry Sonjaya, Bambang Purwantara, Raden Iis Arifiantini, Muhammad Agil, Berlin Pandapotan Pardede, Suyadi Suyadi, Wike Andre Septian, Daud Samsudewa, Erni Damayanti, Tulus Maulana, Syahruddin Said

**Affiliations:** 1 Department of Animal Production, Faculty of Animal Science, Hasanuddin University, Makassar, 90245, Indonesia; 2 Research Center for Applied Zoology, National Research and Innovation Agency (BRIN), Bogor, 16914, Indonesia; 3 Division of Reproduction and Obstetrics, School of Veterinary Medicine and Biomedical Sciences, IPB University, Bogor, 16680, Indonesia; 4 Faculty of Animal Science, Brawijaya University, Malang, 65145, Indonesia; 5 Department of Animal Science, Faculty of Animal and Agricultural Sciences, Diponegoro University, Semarang, 50275, Indonesia

**Keywords:** Bali cattle, embryos, *in vitro*, semen quality

## Abstract

**Background and Aim:**

Fertility is crucial for enhancing the efficiency of livestock production, as it directly impacts the reproductive rates. A comprehensive understanding of the relationship between sperm quality and embryo development is key to optimizing reproductive outcomes and improving the quality of livestock. This study analyzed the developmental competence of *in vitro* embryos recovered from Bali cattle with normal or poor sperm motility.

**Materials and Methods:**

Nine bulls with normal fresh semen (NFS) or poor fresh semen (PFS) motility were ejaculated for semen. Semen ejaculates, including volume, motility, and sperm concentration, were evaluated immediately after collection to measure the quality of the fresh semen. Frozen semen was evaluated using computer-assisted semen analysis (CASA) for motility, progressive sperm motility, distance curve path, distance curve linear, distance straight line, average path velocity, curvilinear velocity, linear velocity, straightness (STR), linearity of forward progression (LIN), wobble, and average lateral head displacement (ALH). Bull groups were used to determine *in vitro* embryo cleavage ability after fertilization of Bali cattle. Ovaries of Bali cattle were collected by slicing, and only cytoplasmic oocytes with compact cumulus cells were used in this study. The oocytes were matured, and *in vitro* fertilization was performed using fertilization media with a final sperm concentration of 1.5 × 10^6^ spermatozoa/mL. After 48 h, the embryo cleavage ability of the cultured oocytes was evaluated.

**Results:**

There were significant differences in motility values between the NFS and PFS groups; however, there were no significant differences in the volume or sperm concentration. There was a significant difference in the LIN value between the groups but no significant differences in other CASA parameters. There were no significant differences in the cleavage rate and morula between the groups, but a positive correlation was observed between the cleavage rate and the morula and between the morula and ALH. A significant negative correlation was observed between the cleavage rate and STR and between the morula and STR; no significant differences were observed for other variables.

**Conclusion:**

Despite variations in sperm characteristics, both normal and poor sperm motility demonstrated comparable *in vitro* embryonic development competence. These findings provide important insights into the fertility potential of Bali bulls, providing valuable information that can enhance selection strategies to improve the quality of livestock production.

## Introduction

Fertility is a fundamental aspect of livestock reproduction, and improving breeding efficiency remains a primary objective for conserving and improving valuable animal populations. Semen quality determines the success of artificial insemination (AI) [1, 2]. AI is an early-stage biotechnological breakthrough that improves livestock’s genetic quality, especially cattle [3]. Existing evidence indicates that the success rate of AI remains relatively low; bulls in Indonesia have fertility levels below 60% [4].

Fertility relies on multiple interconnected factors other than sperm motility and may require a comprehensive mapping from genotype to phenotype for each individual [5]. Sperm motility exhibits considerable variability and accurately characterizing this fundamental variability is challenging. Furthermore, sperm undergo capacitation in the oviduct, which alters several phenotypic traits, including motility patterns [6]. Semen obtained from bulls used for this purpose is typically frozen and acquired from AI centers [7]. Several frequently used characteristics to assess fertility include (i) the number of offspring per delivery (litter size) [8, 9], (ii) the percentage of inseminated females that do not return to estrus (non-return rate), (iii) the percentage of inseminated females that become pregnant (conception rate) [10–12], and (iv) the percentage of inseminated females that successfully reach farrowing (farrowing rate) [13]. In addition, the selective pressures imposed by farms and AI centers on animals can significantly restrict our ability to comprehend the role of natural variation in sperm motility in the overall fertility of these productive species [14].

In recent years, cattle-assisted reproductive technologies, including the *in vitro* production of embryos [15], have made significant advancements. *In vitro* production enables greater rates of genetic improvement by enhancing the reproduction of superior females [16], early embryos, and embryo quality [17]. Assisted reproductive technology, including *in vitro* production of embryos through processes such as maturation, fertilization, and culture, represents a technological solution employed to address issues related to infertility in cattle [18, 19]. *In vitro* fertilization (IVF) can generate a substantial number of embryos at various developmental stages, and the *in vitro* method is useful to assess the potential fertility of bulls based on the analysis of semen [20]. The genetic contributions of both male and female significantly influence the development of embryo. The quality of semen alone cannot guarantee the successful fertilization of oocyte. Therefore, the necessity for *in vitro* validation arises. This *in vitro* validation process enables a comprehensive evaluation of the ability of bull sperm to fertilize oocytes within controlled parameters. IVF can serve as a method to assess a bull’s field fertility based on the cleavage rate and blastocyst formation [21–24].

This study aimed to evaluate the developmental competence of *in vitro* embryos recovered from Bali cattle with normal or poor sperm motility, which can improve the selection and success of *in vitro* technologies. This research will support the identification of superior bulls for semen production at AI centers in Indonesia.

## Materials and Methods

### Ethical approval

The Animal Ethics Commission of Hasanuddin University approved this study (approval number: 302/UN4.6.4.5.31/PP36/2021).

### Study period and location

This study was conducted from March to September 2023. Ovaries were collected from Tamangapa slaughterhouse, Makassar, Indonesia. IVF was evaluated at the Laboratory for *In Vitro* Embryo Production, Hasanuddin University. Semen from the Regional Artificial Insemination Center (RAIC) in South Sulawesi was used. Sperm samples were evaluated at the Genomic Laboratory, National Research and Innovation Agency, Bogor, Indonesia.

### Experimental design and animal care

A total of 135 pairs (1,822 oocytes) of Bali cattle ovaries were obtained from the Tamangapa Slaughterhouse, Makassar. Semen was collected from nine bulls belonging to the South Sulawesi Regional Artificial Insemination Center (RAIC), with ages ranging from 5 to 10 years. Grouping was performed using secondary data from 2021 and 2022. Bulls were maintained according to the standard operating procedures of the South Sulawesi RAIC. Bali bulls were individually kept in 2.5 × 2 m cages equipped with feeding and drinking containers. All bulls were fed with 10% fresh forage and 2 kg concentrate of total body weight twice a day, once in the morning and once in the evening, and water was provided *ad libitum*.

### Frozen-thawed semen

Frozen semen from 2022 bulls was used in this study. Frozen semen was thawed in a water bath at 37°C for 30 s. The straw containing the semen was then dried and the semen was removed and placed in the Eppendorf tube by cutting the plugs of the manufacturer and laboratory on both ends of the straw. Subsequently, the tube containing the semen was placed in a water bath at 37°C for further evaluation.

### Computer-assisted semen analysis (CASA)

Sperm motility was evaluated by placing semen (5 μL) on a glass slide and covering it with a glass cover. Sperm motility was evaluated using CASA with Sperm Vision 3.7 (Minitub, Tiefenbach, Germany). Parameters measured included total motility (%), progressive motility (%), distance average path (DAP: μm/s), distance curve linear (DCL: μm/s), distance straight curve linear (DSL: μm/s), average path velocity (VAP: μm/s), velocity curve linear (VCL: μm/s), velocity straight line (VSL: μm/s), straightness (STR: VSL/VAP), linearity of curve linear (LIN: VSL/VCL), wobble (WOB). The beat cross frequency (BCF: Hz), measured in Hertz, is the frequency with which the sperm head crosses the middle path of the sperm. Sperm motility was observed under a microscope at a magnification of 200×, and as many as ten fields were examined with values ranging from 0% to 100%. The average values for each parameter were determined using approximately 1000 spermatozoa sample [25].

### Acrosomal status

**Figure-1:**
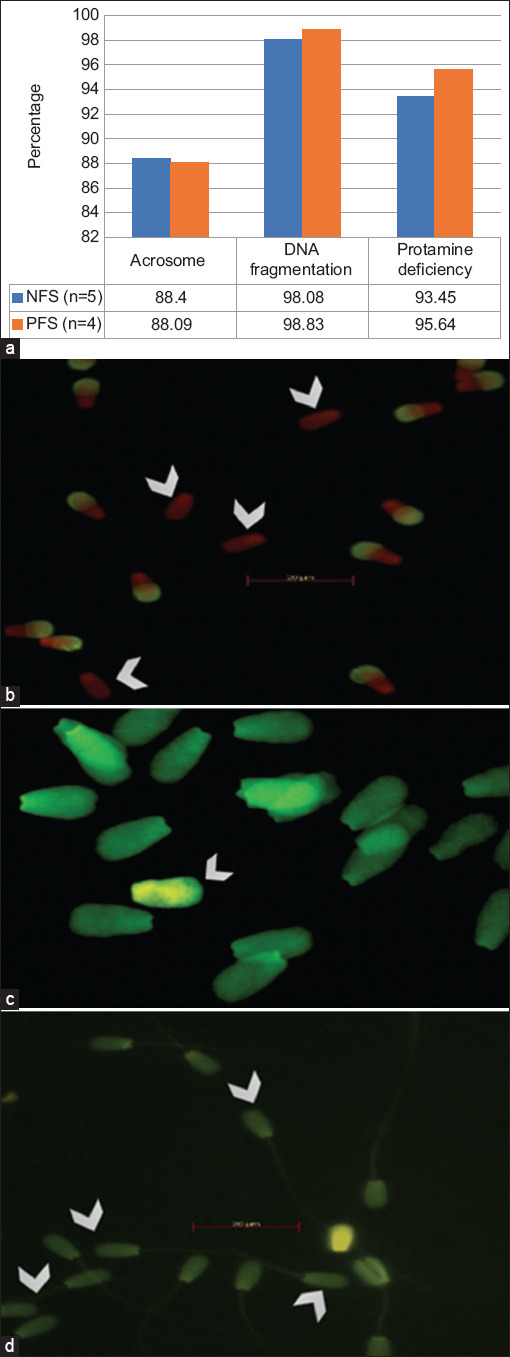
(a) Percentage of crosome integrity, DNA fragmentation, and protamine deficiency of Bali bull with normal and poor sperm motility. (b) Sperm acrosome integrity assessment by FITC−PNA staining; sperm no FITC−PNA staining−sperm with loss−acrosome (arrow). (c) DNA fragmentation assessment by acridine orange staining; sperm with green to yellow fluorescence in the head−sperm with DNA fragmentation (arrow). (d) Sperm protamine deficiency assessment by chromomycin A3; sperm with dull green round−headed sperm cells show protamine deficiency (arrow). Different letters within the same variable indicate significant difference (p < 0.05).

Acrosomal status was evaluated using peanut agglutinin lectin (FITC-PNA; Sigma, St. Louis, MO, USA). The samples were spread onto glass slides, fixed in 96% ethanol at room temperature for 10 min, and air-dried. Subsequently, 30 μL of a 100 μg/mL PNA solution was added and incubated for 30 min at 37°C. Samples (5 μL [1 μg/μL]) were stained with propidium iodide (Sigma–Aldrich, USA) and were incubated for 5 min. After incubation, the samples were washed thrice with phosphate-buffered saline (PBS) to remove residual unbound reagents. Samples were then sealed with a cover glass. Subsequently, 200 cells per slide were examined using a fluorescence microscope (AxioPhot Zeiss, Oberkochen, Germany; 490/530 nm excitation filter) equipped with a 380/420 nm excitation/barrier filter. Spermatozoa with intact acrosomes exhibited green fluorescence in the acrosomal region, whereas those with damaged acrosomes exhibited red fluorescence (Figure-1b) [26].

### DNA fragmentation

DNA fragmentation was evaluated using acridine orange (AO) staining in accordance with the protocol described by Said *et al*. [27]. Semen was placed on a glass slide and dried using a Bunsen burner. Dried semen was then fixed in 200 mL of Carnoy’s solution for 2 h, rinsed with distilled water, and air-dried. Subsequently, 250 mL of AO solution was submerged for 5 min, and the samples were rinsed with distilled water. The slides were covered with a cover glass, sealed with clear nail polish, and examined under a fluorescence microscope (Figure-1c).

### Protamine deficiency

Protamine deficiency was evaluated using the chromomycin A3 (CMA3) fluorescent technique based on the method described by Baharun *et al*. [28]. Semen was fixed in 200 mL of Carnoy’s solution for 10 min at 4°C, followed by thorough washing with PBS and air drying. Subsequently, 30 μL of CMA3 solution was carefully applied to a glass slide and incubated for 20 min at 37°C. Subsequently, it was thoroughly washed with McIlvaine’s buffer (17 mL 0.1 mol/L citric acid mixed with 83 mL 0.2 mol/L Na_2_HPO_4_ and 10 mmol/L MgCl_2_, pH 7.0), air-dried, glued with clear nail polish, and examined under a fluorescent microscope (Figure-1d).

### Oocyte collection and selection

The ovaries obtained from the slaughterhouse were transported to the laboratory using transport media (0.9% NaCl solution, Sigma–Aldrich, USA) supplemented with gentamicin (Sigma–Aldrich, USA). The oocytes were collected by the slicing method.

### *In vitro* maturation

The collected oocytes were washed with PBS (Gibco by Life Technologies, USA) and supplemented with 0.2% bovine serum albumin (BSA; Sigma–Aldrich). Oocytes were matured using M199 medium (Gibco by Life Technologies) supplemented with 0.3% BSA, 10 IU/mL of follicle-stimulating hormone (Intergonan^®^, Intervet GmbH, Unterschleissheim, Germany), 10 IU/mL of human chorionic gonadotrophin (Chorulon, Intervet International BV, Boxmeer, Holland), and 50 μg/mL of gentamicin (Sigma–Aldrich) [29]. Maturation was performed in droplets (80 μL/drop) and covered with mineral oil (Sigma Chemical Co. St. Louis MO, USA) in a 5% CO_2_ incubator at 38.5°C for 24 h [30].

### *In vitro* fertilization

Semen was obtained from the normal fresh semen (NFS, n = 5) and poor fresh semen (PFS, n = 4) bull groups. After thawing for 30 s at 37°C, the semen was centrifuged for 5 min at 1500 rpm to separate the plasma and spermatozoa. Semen was added to the fertilization medium to achieve a final spermatozoa concentration of 1.5 × 10^6^ cells/mL [31]. Subsequently, four drops, each containing 80 μL, were placed in a Petri dish covered with mineral oil (Sigma Chemical Co.) and allowed to equilibrate for 30 min. The matured oocytes were inserted into IVF media [32] and kept in an incubator at 38.5°C with 5% CO_2_ for 5–6 h.

### *In vitro* cultures

After 5–6 h of fertilization, the oocytes were washed twice with Charles Rosenkrans (CR1aa) culture medium (0.4 mM sodium pyruvate, 5 mM lactate, 5% calf serum, 20 µg/mL L-glutamic acid, 100 IU/mL penicillin, 0.1 µg/mL streptomycin sulphate) (modified from the procedure described by Somfai *et al*. [33] and Sagirkaya [34]) and then transferred in a drop of 80 μL of CR1aa culture medium. The samples were supplemented with 5 mg/mL of BSA (Sigma–Aldrich) and 2.5% fetal bovine serum (Sigma–Aldrich) and covered with mineral oil (Sigma Chemical Co.). Subsequently, the media were incubated at 38.5°C in 5% CO_2_ [35]. The developing embryos were then cultured for 48 h and evaluated.

### Statistical analysis

Means and standard errors of means were calculated for all parameters. Statistical differences among bulls were determined using a one-way analysis of variance with Duncan’s multiple range test using SPSS ver. 25.0 (IBM Corp., Armonk, NY, USA). Pearson correlation analysis and linear regression were used to determine the correlation between kinematic CASA and embryo development. All tests used a significance level of p < 0.05.

## Results

### Quality of fresh and frozen sperm from Bali bulls with normal and poor sperm motility

The semen volume percentage was 6.26 ± 1.36 mL in the NFS group and 6.08 ± 1.29 mL in the PFS group. The percentage of fresh semen motility in the NFS group (70.10 ± 0.71%) was significantly higher than that in the PFS group (64.32 ± 2.05%) (p < 0.05). The concentration in the NFS group (939.98 × 10^6^/mL) was lower than that in the PFS group (945.81 10^6^/mL), and there were no significant differences in the volume and concentration between the NFS and PFS groups (p > 0.05) (Table-1).

**Table-1:** Fresh and frozen sperm quality of Bali bulls.

Parameter	Bull	

	NFS (n = 5)	PFS (n = 4)
Volume (mL)	6.26 ± 1.36	6.08 ± 1.29
Motility (%)	70.10 ± 0.71^a^	64.32 ± 2.05^b^
Concentration×10^6^/mL	939.98 ± 1.503	945.81 ± 1.779

Different letters within the same variable indicate significant difference (p < 0.05). NFS=Normal fresh semen, PFS=Poor fresh semen

Table-2 shows a significant difference (p < 0.05) between the LIN values, which were higher in the PFS group (52.00 ± 1.96%) than in the NFS group (48.17 ± 1.92%) (p < 0.05). However, there were no significant differences in the other CASA parameters between the NFS and PFS groups (p > 0.05). The correlation between sperm kinematics and embryo development competence was further analyzed.

**Table-2:** Kinematic sperm of Bali bulls with normal and poor sperm motility.

Parameter	Bull	

	NFS (n = 5)	PFS (n = 4)
Total motility (%)	47.17 ± 3.46	43.94 ± 1.26
Progressive motility (%)	38.61 ± 2.92	39.04 ± 1.81
DAP (μm/s)	27.70 ± 1.06	33.07 ± 6.21
DCL (μm/s)	42.93 ± 8.42	48.24 ± 10.82
DSL (μm/s)	18.91 ± 1.30	2.88 ± 5.67
VAP (μm/s)	67.45 ± 2.82	75.38 ± 10.98
VCL (μm/s)	94.98 ± 3.65	107.23 ± 15.36
VSL (μm/s)	46.43 ± 3.07	55.55 ± 9.20
STR (%)	66.79 ± 4.53	74.33 ± 2.83
LIN (%)	48.13 ± 1.92^a^	52.00 ± 1.96^b^
WOB (%)	70.33 ± 0.41	69.75 ± 1.79
ALH (μm)	5.54 ± 0.37	5.07 ± 0.47
BCF (Hz)	22.07 ± 1.08	27.21 ± 3.80

Different letters within the same variable indicate significant difference (p < 0.05). NFS=Normal fresh semen, PFS=Poor fresh semen, DAP=Distance curve path, DCL=Distance curve linear, DSL=Distance straight curve linear, VAP=Average path velocity, VCL=Curvilinear velocity, VSL=Straight linear velocity, STR=Straightness, LIN=Linearity of forward progression, WOB=Wobble, ALH=Average lateral head displacement, BCF=Beat cross frequency

Frozen semen quality in the NFS and PFS groups was evaluated based on acrosome integrity, DNA fragmentation, and protamine deficiency, as presented in Figure-1. No significant differences were observed in acrosome integrity or DNA fragmentation (p > 0.05) between the NFS and PFS groups. The average acrosome integrity value was higher in the NFS group (88.40%) than in the PFS group (88.09%). Furthermore, DNA fragmentation was significantly lower in the NFS group (98.08%) compared with the NFS group (93.45%) and significantly lower (p > 0.05) compared with the PFS group (95.64%).

### Development competence of Bali cattle embryos with either normal or poor sperm motility

**Figure-2:**
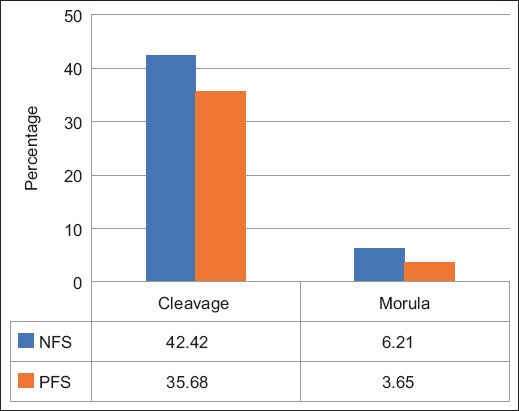
Mean ± standard error of the mean of cleavage and morula rates by group. Legend: Different letters within the same variable indicate significant difference (p < 0.05).

**Figure-3:**
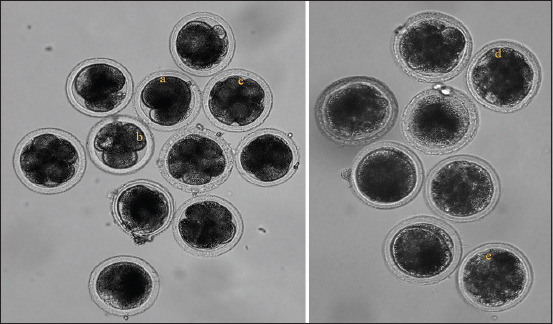
*In vitro* development of Bali cattle embryos cultured (a) 2 cell, (b) 4 cell, (c) 8 cell, (d) 16 cell, and (e) morula (magnification 100×).

There was no significant difference in the developmental competence of Bali cattle embryos between the NFS and PFS groups (p > 0.05). The cleavage percentage was 42.42% in the NFS group and 35.68% in the PFS group, whereas the percentage of embryos reaching the morula stage was 6.12% in the NFS group and 3.65% in the PFS group (Figure-2). In Figure-3, the *in vitro* development of Bali cattle embryos cultured, depicting stages from 2 cell, 4 cell, 8 cell, 16 cell, and morula, is presented.

### Correlation between kinematic sperm motility and development competence of Bali cattle embryos with normal and/or poor sperm motility

A positive correlation was observed between the cleavage rate and morula (p < 0.01) and between morula and average lateral head displacement (ALH) (p < 0.05). In addition, a negative correlation was observed between the division rate and STR (p < 0.05), morula and STR (p < 0.05). Some correlations were not statistically significant (Table-3).

**Table-3:** Correlation between kinematic sperm and embryos development competence of Bali cattle embryos with either normal or poor sperm motility.

Parameter	Cleavage	Morula	Motility	Progressive	VAP (µm/s)	VCL (µm/s)	VSL (µm/s)	STR (%)	LIN (%)	ALH (µm)	BCF (Hz)
Cleavage	1	0.827**	0.308	−0.264	−0.440	−0.556	−0.501	−0.716*	−0.3.90	−0.730*	0.594
Morula		1	0.662	−0.177	−0.432	−0.499	−0.524	−0.790*	−0.570	0.694*	−0.601
Motility			1	0.611	−0.447	−0.336	−0.549	−0.811**	−0.837**	0.505	−0.601
Progressive				1	0.279	0.311	0.158	−0.144	−0.248	−0.189	0.188
VAP (mm/s)					1	0.943**	0.975**	0.694*	0.751*	−0.678*	0.942**
VCL (mm/s)						1	0.959**	0.705*	0.673*	−0.565	0.904**
VSL (mm/s)							1	0.792*	0.840**	−0.673*	0.971**
STR (%)								1	0.863**	−0.745*	0.859**
LIN (%)									1	−0.683*	0.879**
ALH (mm)										1	−0.787*
BCF (Hz)											1

* A significant correlation (p < 0.05).

** A significant correlation (p<0.01). VAP=Average path velocity, VCL=Curvilinear velocity, VSL=Straight linear velocity, STR=Straightness, LIN=Linearity of forward progression, ALH=Average lateral head displacement, BCF=Beat cross frequency

## Discussion

### Semen quality in Bali bulls with either normal or poor sperm motility

Testicular thermoregulation and hormonal regulation can influence semen volume [36]. The semen volume in this study did not differ between the NFS and PFS groups and was consistent with previously reported volumes of 6.32–6.33 mL [36]. Semen volume, motility, and concentration can influence sperm quality and fertility rate [37].

Motility is a primary indicator of sperm quality and fertilization ability [38]. In this study, fresh semen sperm motility was 70% in the NFS group, whereas it was ≥70% in the PFS group. The average sperm motility in this study was lower than that reported by Indriastuti *et al*. [36], which was >80%. Sperm motility is influenced by various factors, such as age, individual male characteristics, season, and temperature [3, 37, 39, 40]. Variations in male sperm motility are also influenced by changes in mitochondrial function in the production of adenosine triphosphate [36]. In addition, the condition of the reproductive system, such as testes, epididymis, and reproductive tract, influences sperm motility.

Sperm concentration was greater in the PFS group than in the NFS group. Although sperm concentration is commonly associated with semen volume, in this study, the semen volume was higher in the NFS group, whereas sperm concentration was lower (Table-1). These results are in accordance with a previous report that semen volume is negatively correlated with sperm concentration [40, 41]. Sperm concentration increases with sexual development, maturity, testicular size, and semen collection frequency [39, 41, 42].

The progressive percentage of motility in the NFS and PFS groups conformed to the requirements of SNI 4869-1:2017 for frozen bovine semen, with a minimum total sperm motility of 40% [43]. The freeze–thaw process can reduce sperm motility by 26.2%–60% [44]. A higher percentage of progressive motility enhances the sperm’s capacity to fertilize oocytes, producing zygotes, which can be evaluated using CASA. The CASA method is a more efficient approach to assess sperm quality because it can accurately and comprehensively measure sperm fertility parameters [45]. To assess the fertility potential of bulls, kinematic parameters such as velocity straight line (VSL), average path velocity (VAP), velocity curve linear (VCL), LIN, ALH, STR, and BCF have been evaluated [46–48].

There were no significant differences in sperm kinematics between the NFS and PFS groups except for LIN (Table-2). Most sperm kinematics in the PFS group were higher than those in the NFS group, except for DSL, WOB, and ALH, which were higher in the NFS group than in the PFS group. A high LIN value in sperm indicates more regular and coherent movements in a straight path, which could potentially serve as an indicator of sperm quality when reaching and binding to an egg during the fertilization process.

Inanç *et al*. [48] reported that the VCL, VAP, and VSL values required for spermatozoa to penetrate the ovum were >70 m/s for VCL and >45 m/s for VAP and VSL. In this study, the VAP, VCL, and VSL values were 67.45 and 75.38 μm/s, 94.98 and 107.23μm/s, 46.43 and 55.55 μm/s, respectively, for the NFS and PFS groups, respectively, indicating that the sperm movement was optimal for reaching and penetrating the egg. Sperm kinematic assessment in this study is consistent with the findings reported by Hasbi *et al*. [41] in horned and polled Bali cattle. Higher VAP and VCL values will increase the proportion of sperm that show hyperactive movements, thereby increasing IVF rates [49].

The findings of this study indicate that the NFS and PFS groups showed good acrosome integrity and DNA fragmentation. Protamine deficiency (Figure-1a) was significantly different between the NFS (93.45%) and PFS (95.64%) groups. High levels of protamine deficiency in sperm can affect the sperm’s ability to fertilize an egg, potentially resulting in failure of the fertilization process.

DNA fragmentation and parameters of sperm quality, fertilization, and embryo development are caused by extrinsic factors and their effector mechanisms that affect fertility levels [50]. Ribas-Maynou *et al*. [51] have reported that increased DNA fragmentation in bovine sperm is associated with morphological abnormalities. However, Otero *et al*. [52] reported a negative correlation between sperm motility parameters and several kinematic parameters with DNA fragmentation. Several studies have reported increased DNA fragmentation caused by age [53], testicular temperature [50], cryopreservation, and sperm storage temperature [50, 54].

### Development competence of Bali cattle embryos with either normal or poor sperm motility

Different percentages of cell division rate were caused by different quality of sperm used in the fertilization process. The NFS group was able to fertilize egg cells and support embryo development. The quality of sperm is influenced by various factors, including motility. Sperm motility increases successful fertilization and cell division during the early stages of embryo development. Before fertilization, the oocyte extends its polar body forward and prepares for fertilization, whereas the sperm becomes hyperactive and ready to fertilize the oocyte [55]. Two main parameters are related to the rate of embryo cleavage: the first cleavage and the percentage of embryos that demonstrate a lag phase, which is influenced by vitrification and partial denudation [56].

A low cleavage rate may also be due to polyspermy. Although some sperm can reach the nucleus of the egg and combine their genetic material, there is an imbalance that prevents the development of the embryo and leads to death. High polyspermy is also a challenging issue in *in vitro* embryo production in pigs [57], whereas in bovine *in vitro* fertilization, high polyspermy is considered a less common problem with an unclear etiology [58]. Despite being regulated by the oocyte [59], strategies implemented at the sperm level have successfully reduced the occurrence of polyspermy [60]. To the best of our knowledge, this is the first study to report that the ≥70% sperm motility group was better at penetrating the oocyte *in vitro* than the ≤70% sperm motility group. Low motility results in fewer sperm reaching the egg [61]. Although the movement of spermatozoa is supported by smooth muscle contractions in the female genital tract [62], high sperm motility is crucial for navigating the strong turbulence near the cilia of the tube wall and reaching the center of the fallopian tube lumen. In addition, low sperm motility leads to delayed fertilization, resulting in older and less competent oocytes at the time of fertilization [61].

Fu *et al*. [63] reported that semen and oocyte quality significantly influence fertilization and cleavage rates. Notably, there are variations in oocyte development among cows, with prepubertal and adult cow oocytes resulting in 10%–15% and ~30% of embryo development, respectively [64]. Ismirandy *et al*. [65] reported a relatively low success rate of embryo transfer using fresh and frozen Bali cattle embryos that are produced through IVF techniques at 40% for fresh embryos and 12.5% for frozen embryos. A previous study has reported that early cleavage produces a higher rate of embryo development [66], and the transfer of embryos with early cleavage can increase the percentage of successful pregnancies [63, 67]. Ensuring the availability of highly motile bull sperm is crucial for the success of *in vitro* fertilization [68].

Furthermore, the correlation between morula and ALH showed that the sperm quality of the NFS and PFS groups was proficient in the fertilization process and embryo development. ALH measures the amplitude of lateral movement of the sperm head and is used to measure sperm motility and movement ability.

## Conclusion

In this study, we found that despite variations in sperm characteristics, both normal and poor sperm motility demonstrated comparable *in vitro* embryonic development competence. These findings provide important insights into the fertility potential of Bali bulls and valuable information that can enhance selection strategies to improve the quality of livestock production.

## Author’s Contributions

HH and HI: Conceived and designed the study. HI, HS, SS, BP, BPP, RIA, and MA: Performed the study. HI, ED, TM, DS, WAS, and SyS: Data curation, validation, formal analysis, visualization, reviewed and edited the manuscript. HH, HI, and DS: Drafted the manuscript. All authors have read, reviewed, and approved the final manuscript.
